# Nifedipine Protects INS-1 β-Cell from High Glucose-Induced ER Stress and Apoptosis

**DOI:** 10.3390/ijms12117569

**Published:** 2011-11-07

**Authors:** Yao Wang, Lu Gao, Yuan Li, Hong Chen, Zilin Sun

**Affiliations:** 1 Department of Endocrinology, Zhongda Hospital, Institute of Diabetes, Southeast University, No.87 Dingjiaqiao Road, Nanjing, Jiangsu 210009, China; E-Mails: yaowang803@126.com (Y.W.); yuanlizhongda@126.com (Y.L.); hongchenzhongda@yeah.net (H.C.); 2 Key Laboratory of Human Functional Genomics of Jiangsu Province, Clinical Diabetes Centre of Jiangsu Province, Nanjing Medical University, Nanjing 210029, China; E-Mail: gaolu_nj@126.com

**Keywords:** nifedipine, Ca^2+^ homeostasis, β-cell, endoplasmic reticulum stress, apoptosis, high glucose

## Abstract

Sustained high concentration of glucose has been verified toxic to β-cells. Glucose augments Ca^2+^-stimulated insulin release in pancreatic β-cells, but chronic high concentration of glucose could induce a sustained level of Ca^2+^ in β-cells, which leads to cell apoptosis. However, the mechanism of high glucose-induced β-cell apoptosis remains unclear. In this study, we use a calcium channel blocker, nifedipine, to investigate whether the inhibition of intracellular Ca^2+^ concentration could protect β-cells from chronic high glucose-induced apoptosis. It was found that in a concentration of 33.3 mM, chronic stimulation of glucose could induce INS-1 β-cells apoptosis at least through the endoplasmic reticulum stress pathway and 10 μM nifedipine inhibited Ca^2+^ release to protect β-cells from high glucose-induced endoplasmic reticulum stress and apoptosis. These results indicated that inhibition of Ca^2+^ over-accumulation might provide benefit to attenuate islet β-cell decompensation in a high glucose environment.

## 1. Introduction

Type 2 diabetes (T2D) is characterized by hyperglycemia and insulin resistance [1,2] and increased level of blood glucose concentration has been proposed to induce β-cell loss in T2D [2–4]. Moreover, studies in cultured β-cell and isolated islets have described that long-term exposure to a high concentration of glucose can trigger β-cell apoptosis [5–7]. In the process, cytochrome *c* release, caspase 3 activation might be the main cause of cell death [3,8]. However, the molecular and cellular mechanisms of high concentration glucose-induced β-cell apoptosis have not been well investigated. Some stress occurrence was involved in high glucose-induced β-cell dysfunction, including oxidation stress, vasoactive cytokines release, barrier function changes, and endoplasmic reticulum (ER) stress [9–11].

Apoptotic ER stress was demonstrated to be critical in high glucose-induced β-cell apoptosis [12,13]. In pancreatic β-cells, ER stress is induced by overloaded chaperons, increased misfolded proteins, ER Ca^2+^ depletion and failure of newly synthesized protein folding [14,15]. Such conditions could activate the unfolded protein response (UPR) that inhibits new protein synthesis, increase folding capacity, and degrade misfolded proteins [16,17]. In this process, a signal pathway such as PKR-like kinase (PERK) was activated. PERK phosphorylates eukaryotic translation initiation factor2α (eIF2α), leads to inhibition of new protein translation [9,14,18] and the proapoptotic transcription factor, C/EBP homologous protein 10 (CHOP), which mediates the lethal effect of PERK signaling, is ubiquitously expressed at a very low level but robustly expressed under ER stress condition [19]. Prolonged ER stress leads to cell apoptosis, in which UPR is not sufficient to deal with accumulated misfolded proteins [17,19].

Consistent Ca^2+^ release from ER stores by calcium influx is the main cause to elicit ER stress to induce cell apoptosis by activating some apoptosis signals such as caspase-3, CHOP [20]. In β-cells, Ca^2+^ is a key regulator not only in cell survival, but also in insulin release. Glucose could activate ATP-dependent potassium channel [21], which leads to membrane depolarization, and voltage-gated l-type Ca^2+^-channels are activated to stimulate intracellular Ca^2+^ release from ER stores, triggering insulin release [21,22]. In T2D, consistent hyperglycemia stimulates sustained elevation of intracellular concentration of Ca^2+^ ([Ca^2+^]*_i_*) for insulin secretion, which activates various Ca^2+^ signals-related apoptosis pathways, including ER stress [20,23,24]. Thus, inhibition of an elevated level of [Ca^2+^]*_i_* might benefit T2D treatment.

To investigate the potential role of Ca^2+^ in high concentrations of glucose-induced INS-1 β-cell apoptosis, nifedipine was utilized for efficacy studies, as one of l-type Ca^2+^-channel antagonists [25]. In this study, we confirmed that Ca^2+^ influx is strongly involved in high glucose-related β-cell apoptosis via ER stress pathway, and nifedipine could protect INS-1 β-cells from high glucose-induced ER stress and apoptosis.

## 2. Materials and Methods

### 2.1. Reagents

All general reagents for cell culture were purchased from GIBCO, USA. Nifedipine, hoschst 33342 and DAPI were from Sigma-Aldrich, USA. The fluorescence dyes Fluo-4/AM were from Invitrogen, USA. Insulin ELISA kit were from Millipore. Rabbit anti-GAPDH, phosphor-eIF2α, eIF2α, caspase 3 and insulin antibodies were purchased from Cell signaling technology, rabbit anti-CHOP (GADD153) antibody were from Santa Cruz Biotechnology. Peroxidase-conjugated Goat anti-rabbit IgG was purchased from Jackson Immuno Research.

### 2.2. Cell Culture

Rat insulinoma cell line INS-1 was obtained from American type culture collection (ATCC). INS-1 cells were cultured in RPMI-1640 medium containing 10% (vv-l) fetal bovine serum (FBS), 5.5 mM glucose, 10 mM HEPES, 100 units/mL penicillin, 100 μg/mL streptomycin and 50 μM β-mercaptoethanol at 37 °C and 5% CO_2_ condition. Before the co-treatment with glucose at different concentration and nifedipine, cells were precultured in low-glucose condition (5.5 mM) overnight. In each glucose concentration, cells were incubated with or without 10 μM nifedipine for indicated time.

### 2.3. MTT Assay

INS-1 cells were seeded in 96-well plates (10, 000 cells per well) and treated as described above. After 24 h cultured, cell viability was determined by using a 3-(4,5-dimethylthiazol-2-yl)-2, 5-diphenyltetrazolium bromide (MTT) assay described previously [26]. The results were shown as relative optical density.

### 2.4. Hoechst 33342 Staining

Apoptotic cells are evaluated by Hoechst 33342 staining. The nuclear of cells are stained by Hoechst 33342 and show blue fluorescence. Compared with normal cells, the nuclei of apoptotic cells have highly condensed chromatin which could be visualized by fluorescence microscopy.

### 2.5. Tunel Staining

Cells were cultured on coverglasses in 12-well plates. After 24 h treatment, the apoptotic cells were stained by tunel staining kit following its protocol, the apoptotic cells were stained by green fluorescence, and all cells were marked with blue fluorescence using DAPI. The apoptotic ratio was calculated as tunnel-positive cells divided by total cell number.

### 2.6. Western Blot Analysis

INS-1 cells were treated as described above, and then cells were lysed by protein extraction kit (Beyotime, CN) according to its protocol. Western blot was performed as previously described [27], the following primary antibodies were used: phosphor-eIF2α (dilutions 1:1000), eIF2α (1:1000), CHOP (1:200), caspase 3 (1:500), GAPDH (1:5000).

### 2.7. Calcium Mobilization Assay

Calcium mobilization assay was performed as described in [28]. In acute calcium influx determination, cells were stimulated by 33.3 mM glucose for 1 min in the presence of 0, 1, 3, 10 μM nifedipine. For long-term experiment, after 6 or 24 h treatment, the medium was removed and cells were pretreated with medium without glucose for 1 h, then INS-1 cells were stimulated with 33.3 mM glucose to measure [Ca^2+^]*_i_*.

### 2.8. Insulin Staining and Glucose Stimulated Insulin Secretion (GSIS) Assay

INS-1 cells were seeded into 96-well plates (30,000 cells per well). After the treatment, cells were fixed for 2 h in 4% paraformaldehyde and then incubated with insulin antibody as described [29], the insulin content in INS-1 cells was captured by fluorescence microscope. In the end of treatment, cells were washed twice with Krebs buffer (118 mM NaCl, 4.7 mM KCl, 1.2 mM KH_2_PO_4_, 1.2 mM MgSO_4_, 4.2 mM NaHCO_3_, 2 mM CaCl_2_), then the cells were incubated with 100 μL Krebs buffer containing 2.5 mM glucose for 30 min. Subsequently, wells were washed twice again and incubated with 20 mM glucose Krebs buffer instead. After 1 h incubation, the supernatant from each well were collected, following, the concentration of insulin was measured by rat insulin ELISA kit according to the manufacturer’s instructions (Millipore).

### 2.9. Statistical Analysis

Data were expressed as the mean ± SD. Two-tailed *t* test was performed and a value of *p* < 0.05 was considered significant.

## 3. Results

### 3.1. Nifedipine Protects INS-1 Cells from High Glucose-Induced Apoptosis

**Figure 1:**
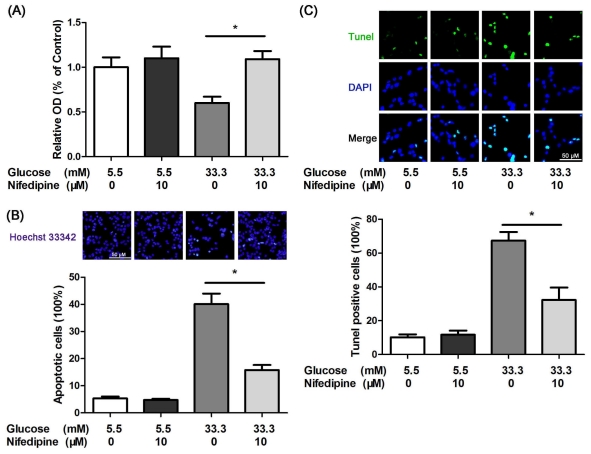
(**A**) Nifedipine prevents high glucose-induced reduction of cell viability in INS-1 cells. 5.5 mM glucose with (black bar) or without (white bar) nifedipine had no change in OD. 33.3 mM glucose (grey bar) significantly reduced the cell viability, 10 μM nifedipine reversed this effect; (**B**) Nifedipine protected INS-1 cells from high glucose-induced apoptosis. Cells were divided into four groups as described above, Hoechst 33342 staining was performed to calculate the apoptotic rate, data was repeated at least three times, scale bar = 50 μm, and referred to all panels; (**C**) Tunel staining of apoptotic cells. Apoptotic cells were marked with green fluorescence, the nuclei of cells are stained by blue fluorescence, Data was repeated at least three times, scale bar = 50 μm, and referred to all panels. Data was shown as mean *±* SE of three independent trails each performed with triplicate samples. The statistics graph shows * *p* < 0.05 compared to 33.3 mM glucose treated group.

High concentration of glucose has been demonstrated to be toxic to cultured β-cells [5–7]. In a concentration of 33.3 mM, glucose inhibited the cell viability compared with 5.5 mM glucose treated group (Figure 1A). However, 10 μM nifedipine suppressed this process significantly, which had no effect on 5.5 mM glucose cultured group (Figure 1A). In addition, nifedipine could not promote cell viability in normal glucose concentration (11.1 mM), but the value of its viability was approximately two times more than other groups (data not shown).

The reduction of cell viability under high glucose-treatment could be involved in increased cell apoptotsis. To determine the apoptotic rate of treated INS-1 cells, Hoechst 33342 staining was performed. The cells incubated with 33.3 mM glucose showed less live cells and highly condensed chromatin in nuclei (Figure 1B). However, 10 μM nifedipine obviously decreased the apoptotic ratio in comparison with high glucose treated cells (Figure 1B). Furthermore, the result of tunel staining indicated that nifedipine prevented high glucose-induced INS-1 cell apoptosis (Figure 1C). Taken together, it suggested that inhibition of calcium influx by l-type-Ca^2+^-channel antagonist could provide benefit to prevent high glucose-induced β-cell apoptosis.

### 3.2. Nifedipine Prevents High Glucose-Induced β-Cell Death Through ER Stress Pathway

**Figure 2:**
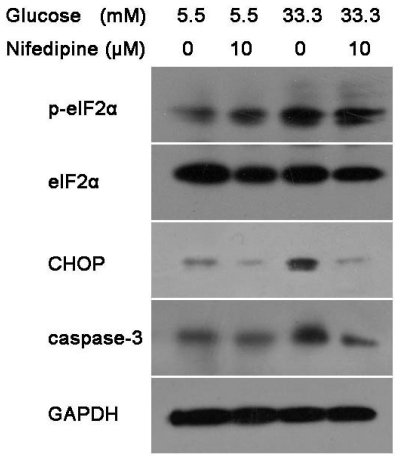
Western blot analysis of protein level of ER stress in INS-1 cells. Cells were divided into four groups as described above. After treatment, cell lysate was collected and subjected to western blot. Each blot was replicated at least three times.

Pancreatic β-cell generates a highly developed ER for Ca^2+^ stores, insulin biosynthesis and secretion [18,30,31]. Thus, high glucose-induced β-cell apoptosis could be closely related to the breakdown of Ca^2+^ homeostasis, which was able to trigger ER stress [32]. To identify whether nifedipine protected INS-1 cells from high glucose-induced apoptosis via ER stress, the expression levels of some proteins involved in ER stress pathway were analyzed by western blot. As depicted in Figure 2, high glucose caused an elevated expression level of p-eIF2α, CHOP and caspase 3 proteins after 24 h treatment. However, these enhanced ER stress markers were reduced by nifedipine (Figure 2), suggesting that l-type-Ca^2+^-channel antagonists could suppress the high glucose-induced ER stress in INS-1 β-cells line.

### 3.3. Nifedipine Improves the High Glucose-Impaired Calcium Homeostasis and GSIS in INS-1 Cells

**Figure 3:**
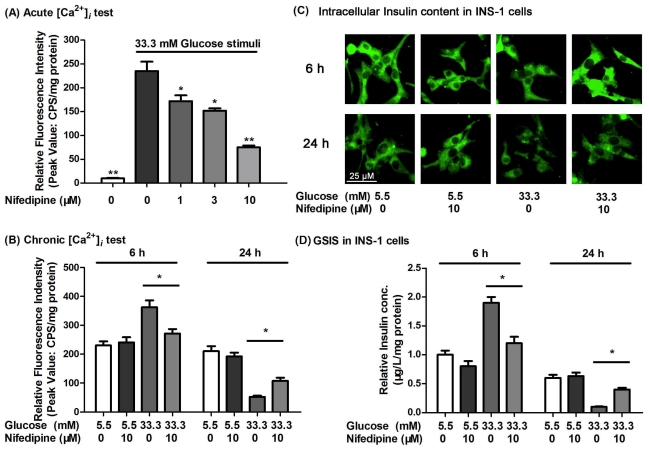
(**A**) The effects of nifedipine and glucose on [Ca^2+^]*_i_* level and glucose stimulated insulin secretion (GSIS). In acute [Ca^2+^]*_i_* determination, nifedipine significant inhibited the calcium influx in 33.3 mM glucose stimulation dosedependently; (**B**) After 6 h treatment, 33.3 mM glucose (dark grey bar) stimulated calcium influx, and 10 μM nifedipine (grey bar) partially prevented this effect. 24 h 33.3 mM glucose treatment impaired the calcium influx in INS-1 cells, but nifedipine partly inhibited this effect; (**C**) The insulin content of INS-1 cells was marked with green fluorescence, 24 h incubation of 33.3 mM glucose obviously reduced the content of intracellular insulin in INS-1 cells. Scale bar = 25 μm, and refers to all panels; (**D**) GSIS in nifedipine and glucose incubated INS-1 cells. After 24 h treatment, 33.3 mM glucose (dark grey bar) impaired GSIS of INS-1 cells, and 10 μM nifedipine (grey bar) partially prevented this effect. * *p* < 0.05 compared to 33.3 mM glucose treated group.

Chronic hyperglycemia is thought to be important in the pathogenesis of T2D [33,34]. Under physiological conditions, an increase in [Ca^2+^]*_i_* in β-cells is of crucial importance in coupling the GSIS [35,36]. Thus, the l-type-Ca^2+^-channel antagonist, nifedipine, might inhibit the sustained increase in [Ca^2+^]*_i_*. We had investigated the effect of nifedipine on the calcium influx in INS-1 β-cells. Nifedipine could dose-dependently inhibit calcium influx in INS-1 cells (Figure 3A). When cells were treated for 6 h, high glucose promoted the activation of calcium influx and GSIS function (Figure 3B,D). However, in long-term incubation, the function of calcium influx was impaired by high glucose, which was protected by nifedipine (Figure 3B). Similarly, high glucose-impaired insulin content and GSIS in INS-1 cells were also protected by nifedipine (Figure 3C,D). It seemed that intracellular calcium homeostasis played an important role in insulin secretion and synthesis. Insulin release in pancreatic β-cells was through the modulation of calcium influx, but the accumulation of excessive calcium would damage cells function, eventually leading to the β-cells apoptosis. Thus, nifedipine could partially prevent high glucose-induced INS-1 cell dysfunction through the regulation of adequate calcium levels. However, the abrogation of l-type-Ca^2+^-channel activation did not completely prevent INS-1 cells from high glucose-induced apoptosis, suggesting that this event may not be totally dependent on calcium homeostasis.

## 4. Discussion

Sustained high glucose *in vivo* may disturb calcium metabolism in islet β-cells [37], which was a possible cause of hyperinsulinism in the development of diabetes mellitus. Calcium homeostasis is also important to the diversity of cellular processes including proliferation and apoptosis [38]. Nifedipine is a dihydropyridine calcium channel blocker, which has been used as antianginal and antihypertensive [39]. Recently, it has been reported that Nifedipine prevented induced apoptosis in endothelial cells and smooth muscle cells [40,41]. The l-type Ca^2+^ channel has been reportedly expressed in INS-1 β-cells [42,43], and β-cells were also thought to be sensitive to calcium channel blockers [44], and Jeffrey *et al*. demonstrated that blocking Ca^2+^ influx with nifedipine could abolish the palmitate-induced ER stress [45]. Accordingly, in obesity or hyperglycemia associated T2D, the sustained elevation of [Ca^2+^]*_i_* is able to generate cell death, and calcium channel antagonists could protect β-cells from palmitate-induced apoptosis [46]. However, the relationship between calcium channel and high glucose-induced cell apoptosis has not been fully understood.

Both free fatty acids (FFA)-amplified insulin secretion and the GSIS itself depend on calcium influx through calcium channels [47,48]. Thus, calcium influx could be a critical mediator not only in high FFA-induced β-cell dysfunction, but also in high glucose-induced impaired β-cells.

In addition, previous studies had shown that high glucose-induced apoptosis occurred through store-operated calcium entry and calcineurin in human umbilical vein endothelial cells and calcium channel blocker (diltiazem) inhibits apoptosis of vascular smooth muscle cell exposed to high glucose concentration [49,50]. Our studies had verified that the calcium channel blocker, nifedipine, could reverse high glucose-induced β-cell dysfunction and apoptosis. As a result of hyperglycemia or hyperlipidemia in T2D, increased β-cell Ca^2+^ flux could play a key role in the failure of β-cells.

During the pre-diabetic state, pancreatic β-cells might trigger a compensatory phase [51,52]. In this phase, an elevated [Ca^2+^]*_i_* could activate increased insulin output to adapt elevated glucose or FFA level [34,52]. However, long-term hyperinsulinemia will exhaust β-cells, and various Ca^2+^ signals related to apoptosis are activated, such as PKR-like kinase (PERK) pathway [9,14]. In our results, PERK phosphorylated eukaryotic translation initiation factor2α led to inhibition of new protein translation [18]. In the process, a proapoptotic transcription factor, CHOP was up-regulated and activated death-related ER stress [20,53]. Finally, caspase 3 was activated and cytochrome *c* release generated cell death.

Nifedipine was reported to inhibit calcium influx [54] and suppressed glucose stimulated insulin secretion [42,54–56] in pancreatic β-cells and islets, but in the long term, exposure to high glucose, the dysfunction of calcium influx and insulin secretion in β-cells occurs. However, Nifedipine might prevent this compensation mechanism, which protects β-cells from glucose-induced dysfunction, and partially due to the regulation of insulin secretion and blocking of calcium-induced apoptotic signal.

In conclusion, high glucose induces β-cell apoptosis which is highly dependent on calcium influx and calcium influx might be of crucial importance in the apoptosis process via ER stress pathway. Our results indicated that inhibition of calcium channel could benefit prevention of cell apoptosis, and suggest that the suppression of β-cell compensation might be beneficial in T2D.
